# Correction: Value sets and the problem of redundancy in value set repositories

**DOI:** 10.1371/journal.pone.0322239

**Published:** 2025-04-04

**Authors:** Sigfried Gold, Harold P. Lehmann, Lisa M. Schilling, Wayne G. Lutters

The hyperlink to the OSF repository in the Data Availability statement is incorrect. The correct hyperlink is: https://osf.io/abtju.

## References

[1] Gold S, Lehmann HP, Schilling LM, Lutters WG. Value sets and the problem of redundancy in value set repositories. PLoS One. 2024;19(12): e0312289. doi: 10.1371/journal.pone.0312289 39652546 PMC11627404

